# Dual Thoracodorsal Nerve Origins With Coexisting Conventional and Accessory Thoracodorsal Arterial Channels: A Cadaveric Case With Additional Axillary Variants

**DOI:** 10.7759/cureus.117177

**Published:** 2026-09-29

**Authors:** Maria Piagkou, George Trikoilis, Alexandros Samolis, Christos Lyrtzis, George Triantafyllou

**Affiliations:** 1 Department of Anatomy, School of Medicine, Faculty of Health Sciences, National and Kapodistrian University of Athens, Athens, GRC; 2 Department of Anatomy, School of Medicine, Faculty of Health Sciences, Aristotle University of Thessaloniki, Thessaloniki, GRC

**Keywords:** intercostobrachial nerve, latissimus dorsi flap, posterior cord, superior thoracic artery, thoracodorsal artery, thoracodorsal nerve

## Abstract

The thoracodorsal artery and nerve are key structures in axillary, breast, and reconstructive surgery. During further dissection of the right axilla of a 78-year-old formalin-embalmed donated male cadaver, two thoracodorsal nerves were identified arising independently from the posterior cord at separate proximal levels. Two arterial channels also coursed toward the thoracodorsal territory: a conventional thoracodorsal artery arising through the subscapular system and an additional thoracodorsal-directed channel arising from a lateral thoracic-thoracodorsal trunk. Because accompanying venous anatomy, terminal intramuscular territories, and functional contributions were not mapped, these findings are described as two independently arising thoracodorsal nerve trunks with conventional and accessory thoracodorsal arterial channels rather than as two complete, functionally independent neurovascular pedicles. An accessory superior thoracic arterial channel and a communication between an intercostobrachial branch and a posterior-cord-derived branch situated between the two thoracodorsal nerve origins were also observed. The same donor and right axilla had previously been reported for other muscular and axillary branching variants; therefore, this report focuses on findings recognized during subsequent deeper dissection and explicitly separates them from previously published anatomy. These observations expand the spectrum of thoracodorsal and axillary variation and emphasize the need to identify unexpected arterial and neural structures during surgery without assuming that close spatial association necessarily represents a duplicated complete pedicle.

## Introduction

The thoracodorsal artery (TDA) and thoracodorsal nerve (TDN) are routinely encountered during axillary, breast, and reconstructive procedures and form the principal vascular and motor supply to the latissimus dorsi (LD). The TDA most commonly arises from the subscapular artery, whereas accessory TDAs or thoracodorsal-directed arterial channels may coexist with a conventional TDA or arise from alternative axillary branches [1-4]. The TDN usually arises from the posterior cord (PC) and divides distally near or within the LD; proximal variation includes accessory branches and separate neural trunks [5-8].

For the present report, an anatomical duplication of the TDN is defined conservatively as two separate nerve trunks with distinct proximal origins from the PC, rather than distal bifurcation of a single common TDN. This definition establishes anatomical multiplicity at origin but does not imply functional independence, separate intramuscular territories, or equivalent motor contribution unless stimulation or intramuscular mapping is performed. Similarly, an accessory thoracodorsal arterial channel is considered a supernumerary vessel that coexists with the conventional subscapular-TDA pathway and courses toward the thoracodorsal/LD territory; a replacement variant would require absence of the conventional TDA. We do not use the term complete neurovascular pedicle for this central finding because we did not map the accompanying venous anatomy.

Additional axillary variations relevant to the present case include unusual superior thoracic arterial patterns [9], variations and communications of the intercostobrachial nerve [10], and contributions of T2 fibers to the brachial plexus [11]. The anatomy of the same donor and right axilla was previously reported in relation to other muscular, neural, and arterial variations [12]. Developmental remodeling of the upper-limb arterial plexus provides a general framework for understanding persistent accessory arterial channels [13]. Variation in the bifurcation and neural pedicle of the thoracodorsal nerve is also relevant to latissimus dorsi flap surgery [14], while considerable morphological variability of the intercostobrachial nerve and its communications has been documented with potential surgical significance [15]. This distinction is particularly relevant after the recent report by Ormston and Lin, who described two neural branches supplying the LD and demonstrated muscle contraction after stimulation of both branches [16]. Their report provides functional evidence that cannot be inferred from gross cadaveric anatomy alone and therefore serves as an important comparator for the present case.

Filippou et al. previously reported the same 78-year-old donor and right axilla in a study focused on a multiheaded coracobrachialis muscle and associated brachial plexus and axillary arterial variations [12]. That publication illustrated the general right axillary branching pattern, one TDA accompanied by a TDN, and four intercostobrachial/lateral cutaneous intercostal branches. The present report is based on subsequent deeper dissection and re-tracing of the preserved axilla. It focuses on the newly recognized separate PC origin of a second thoracodorsal nerve, the additional thoracodorsal-directed arterial channel and its relationship to the lateral thoracic system, and the PC-intercostobrachial communication. Structures previously visible are retained only as anatomical landmarks and are explicitly identified as previously reported.

For contextual comparison, PubMed/MEDLINE was searched through September 20, 2026 using combinations of the terms “thoracodorsal nerve,” “thoracodorsal nerve duplication,” “accessory thoracodorsal nerve,” “thoracodorsal artery,” “accessory thoracodorsal artery,” “superior thoracic artery,” “intercostobrachial nerve,” and “posterior cord”; reference lists and relevant publisher pages were also screened. The search identified reports of accessory TDAs, accessory or variant TDNs, and the recent functionally tested dual TDN branches [3,4,7,8,16]. The purpose of this report is therefore not to claim novelty for any single component, but to document the regional coexistence and precise topographic relationships of multiple thoracodorsal and axillary variants while clearly distinguishing direct observation from interpretation.

## Case presentation

During routine educational dissection of the right axilla of a 78-year-old Greek donated male cadaver fixed in formalin, the axillary neurovascular structures were re-examined after the previously published superficial dissection [12]. No evidence of previous surgery or traumatic distortion of the dissected upper limb was present.

The cadaver was donated to the Anatomy Department, School of Medicine, National and Kapodistrian University of Athens, through the institutional Body Donation (Anatomical Gift) Program after written informed consent. Because this study is a cadaveric report based exclusively on a donated cadaver used for teaching and research, the program's institutional framework did not require separate institutional ethical approval. The donor’s written informed consent authorized the use of the donated body for anatomical education and research and the scientific dissemination of anonymized anatomical findings and images.

The preserved right axilla was dissected progressively from superficial to deep and from proximal to distal. The PC was exposed sufficiently to identify the proximal origins of the relevant branches, and the thoracodorsal-directed nerves were followed distally toward the LD as far as the preserved dissection permitted. The arterial channels were likewise traced proximally to their parent vessels and distally toward the thoracodorsal region. No vascular injection, perfusion study, nerve stimulation, histology, intramuscular nerve mapping, venous mapping, or systematic morphometric protocol had been performed during the original dissection; external diameters, inter-structure distances, and dissected lengths were therefore not retrospectively reported. This limitation guided the conservative terminology used below.

Dissection was performed under direct vision using standard anatomical dissection instruments, including scalpels, fine scissors, forceps, and blunt probes. No optical magnification was routinely used. We performed fine dissection to separate and trace the neural and arterial structures from their proximal origins along their distal courses while preserving their spatial relationships with adjacent axillary structures.

We re-evaluated the relationship with the previous publication before preparing the present report. The general axillary arterial field, one TDA-TDN relationship, and four intercostobrachial/lateral cutaneous intercostal branches had already been illustrated in 2023 [12]. During subsequent deeper tracing, we re-evaluated the vessel previously labeled superficially as LTA3 in relation to the lateral thoracic-thoracodorsal pathway, and additional neural and arterial continuity became apparent. This report does not treat the previously illustrated structures as new findings; novelty is limited to the deeper tracing and newly recognized relationships described in Table 1.

**Table 1:** Transparency of previously published and newly reported findings. TDN, thoracodorsal nerve; TDA, thoracodorsal artery; PC, posterior cord; ICBN, intercostobrachial nerve

Feature	2023 report [12]	Present report	Interpretation
Donor/right axilla	Same 78-year-old male donor; right axilla illustrated	Same preserved axilla re-dissected	Explicitly disclosed
Coracobrachialis and broad AA branching	Primary subject of prior report	Context only	Not claimed as new
One TDA and one TDN	Previously visible/illustrated	Used as landmarks; deeper tracing performed	Not claimed as new structures
Lateral thoracic vessel labeled LTA3	Superficial designation in prior dissection	Re-evaluated after deeper tracing in relation to LTDT/accessory thoracodorsal pathway	Reinterpretation after further dissection
Second independently arising TDN	Not described	Separate PC origin documented	New observation
Additional thoracodorsal-directed arterial channel	Not characterized as such	Traced proximally to the LTDT, from which it arose; conventional TDA preserved	Newly characterized observation
Four ICBN/lateral cutaneous branches	Previously illustrated	Retained for orientation	Not claimed as new
PC-derived branch communicating with ICBN	Not described	Observed between the two TDN origins	New observation
Second superior thoracic arterial channel	Not emphasized	Observed but classified conservatively as accessory channel	Secondary observation

All photographs presented in Figures 1-3 were obtained during the subsequent, more extensive dissection of the preserved right axilla and were specifically acquired for the present report. No photograph or image panel previously published in the 2023 report [12] is reproduced, reused, or adapted in the present manuscript. Previously reported anatomical structures that remain visible in the current images are shown only as landmarks for orientation and for demonstrating their relationship to the newly identified findings.

**Figure 1:**
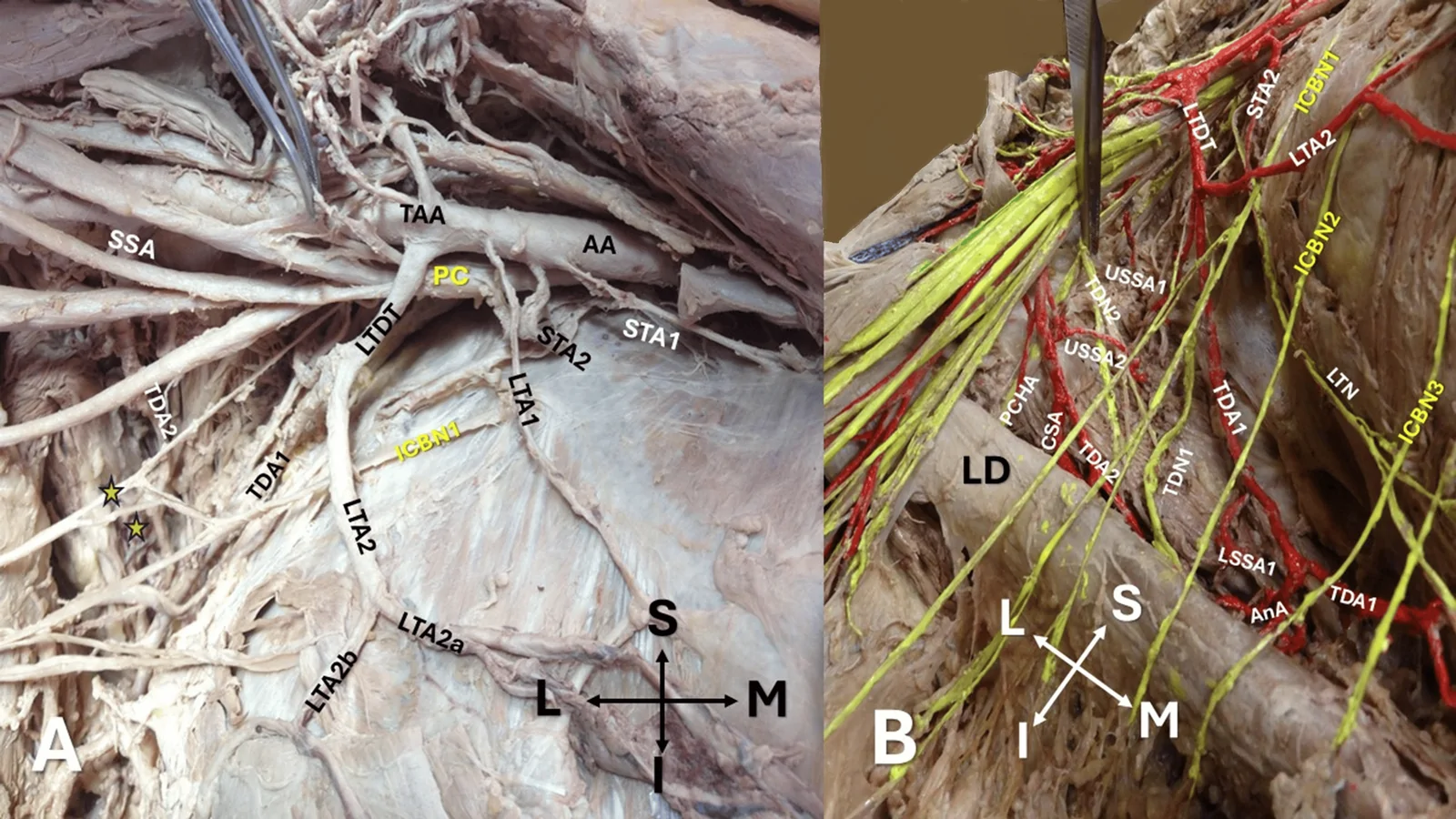
Overall arterial and neural configuration of the right axilla. (A) Unaltered reference view from the present re-dissection showing the proximal axillary arterial branching pattern and the surrounding neural structures. (B) Deeper digitally color-enhanced view highlighting the conventional superior thoracic artery and an additional superior thoracic arterial channel, the conventional thoracodorsal artery (TDA2), and the accessory thoracodorsal arterial channel (TDA1) arising from the lateral thoracic-thoracodorsal trunk (LTDT). Digital enhancement was applied solely to improve visual discrimination of already visible anatomical structures and does not imply an independent terminal perfusion territory. AA, axillary artery; CSA, circumflex scapular artery; ICBN1–3, intercostobrachial/lateral cutaneous intercostal branches; LSSA1, lower subscapular artery; LTA1–2, lateral thoracic arteries; LTA2a–b, branches of LTA2; LTDT, lateral thoracic-thoracodorsal trunk; LTN, long thoracic nerve; PC, posterior cord; PCHA, posterior circumflex humeral artery; SSA, subscapular artery; STA1, conventional superior thoracic artery; STA2, additional superior thoracic arterial channel; TAA, thoracoacromial artery; TDA1, accessory thoracodorsal arterial channel; TDA2, conventional thoracodorsal artery; TDN1–2, thoracodorsal nerve trunks; USSA1–2, upper subscapular arteries, AnA, angular artery; LD-latissimus dorsi muscle. Orientation: S, superior; I, inferior; L, lateral; M, medial.

**Figure 2:**
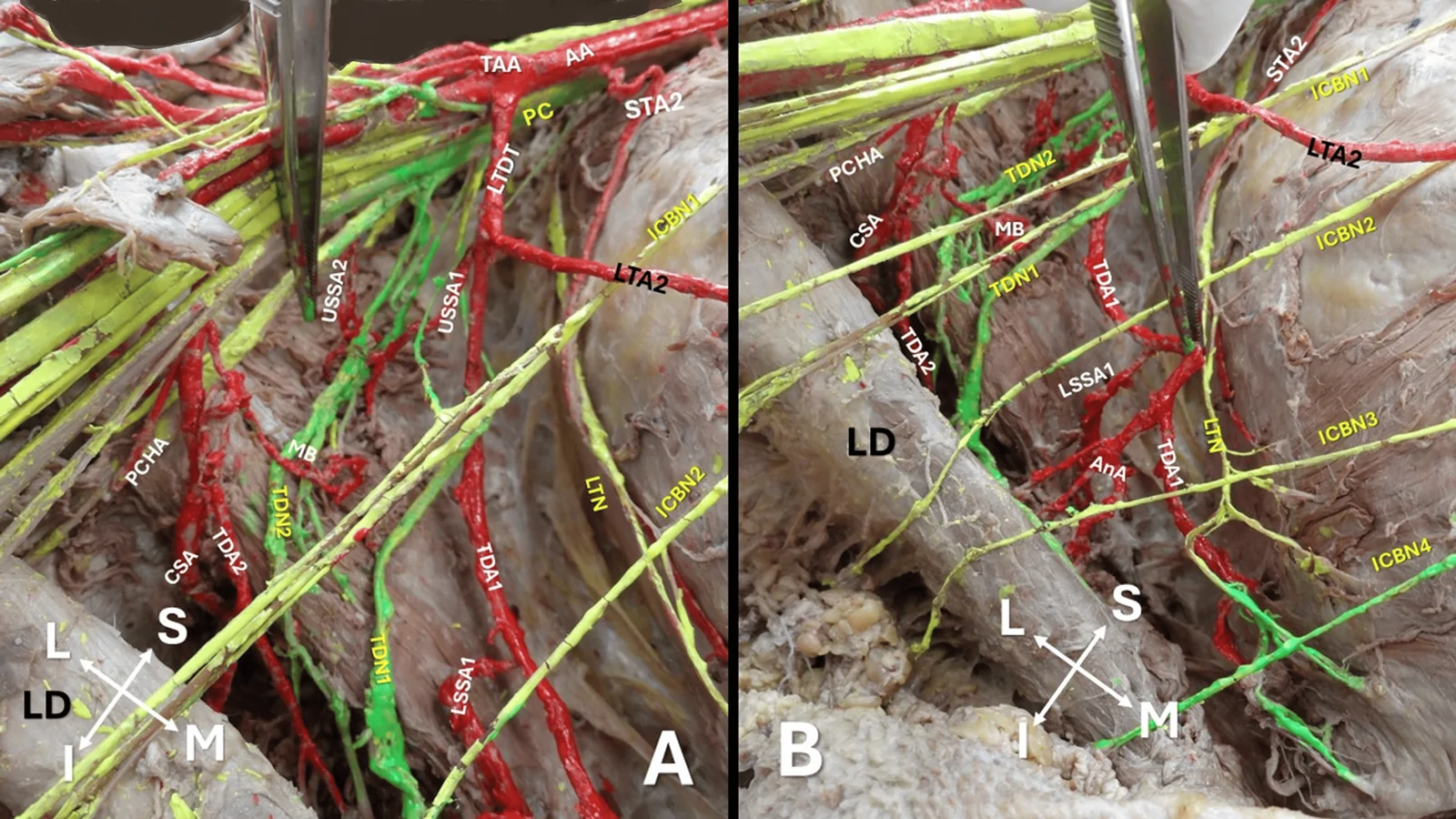
Separate posterior-cord origins and courses of the two thoracodorsal nerve trunks in the right axilla. (A) Digitally color-enhanced dissection demonstrating the posterior cord (PC) and the two thoracodorsal nerve trunks (TDN1 and TDN2), which arise independently at different levels without a common proximal stem. The adjacent conventional thoracodorsal artery (TDA2), accessory thoracodorsal arterial channel (TDA1), lateral thoracic-thoracodorsal trunk (LTDT), and surrounding axillary vessels and nerves are shown for topographic orientation. (B) Deeper view demonstrating the distal courses of TDN1 and TDN2 and their relationships to the two thoracodorsal arterial channels and adjacent intercostobrachial/lateral cutaneous intercostal branches. Digital enhancement was applied solely to improve visual discrimination of already visible anatomical structures. The figure demonstrates separate proximal origins and distal courses of TDN1 and TDN2 but does not establish functional independence or separate intramuscular territories. AA, axillary artery; CSA, circumflex scapular artery; ICBN1–4, intercostobrachial/lateral cutaneous intercostal branches; LSSA1, lower subscapular artery; LTA2, lateral thoracic artery; LTDT, lateral thoracic-thoracodorsal trunk; LTN, long thoracic nerve; MB, muscular branch; PC, posterior cord; PCHA, posterior circumflex humeral artery; STA2, additional superior thoracic arterial channel; TAA, thoracoacromial artery; TDA1, accessory thoracodorsal arterial channel; TDA2, conventional thoracodorsal artery; TDN1–2, thoracodorsal nerve trunks; USSA1–2, upper subscapular arteries, AnA, angular artery; LD-latissimus dorsi muscle. Orientation: S, superior; I, inferior; L, lateral; M, medial.

**Figure 3:**
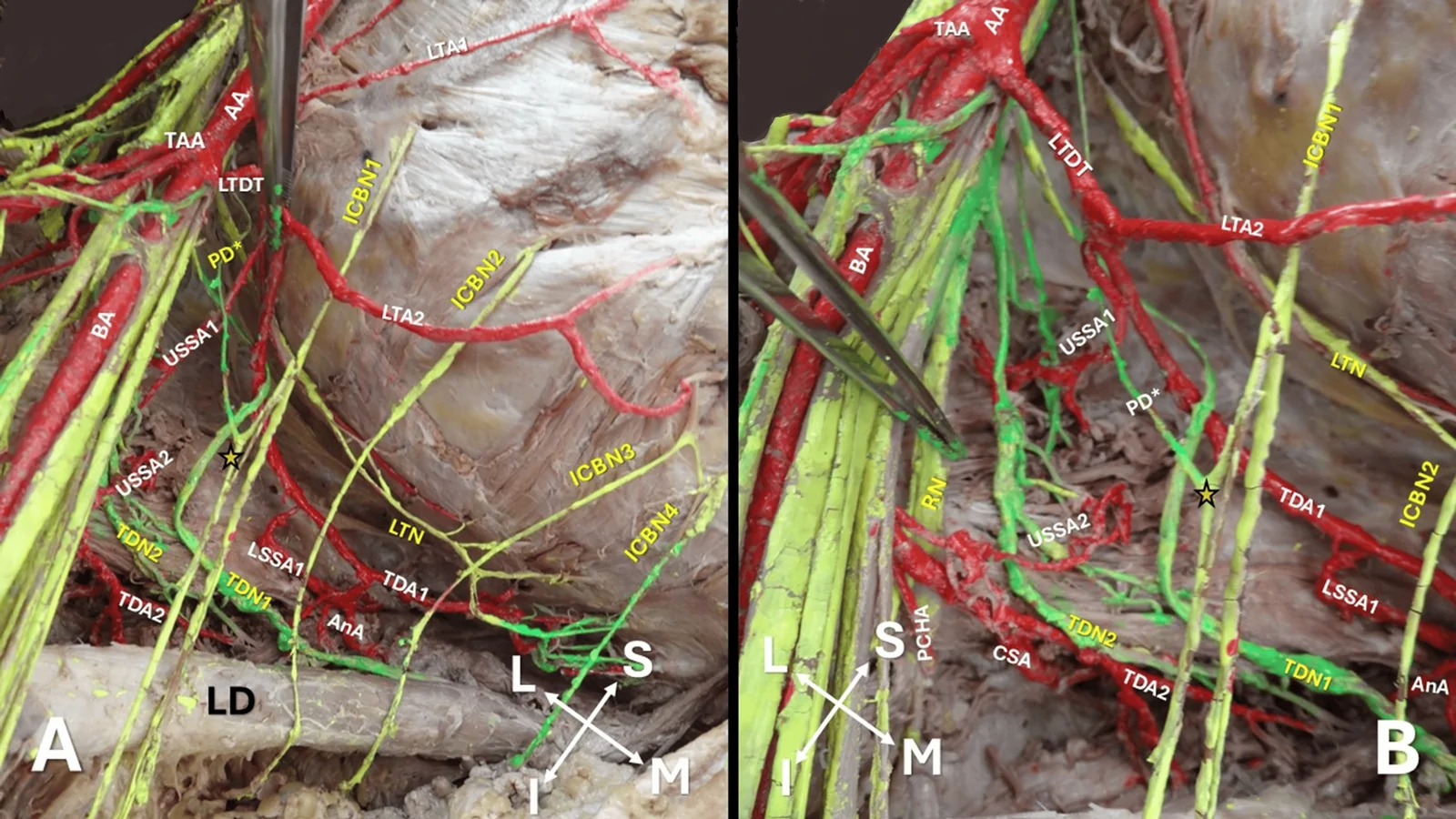
Deeper digitally color-enhanced views of the thoracodorsal arterial and neural structures in the right axilla. (A) Deeper dissection showing the conventional thoracodorsal artery (TDA2), accessory thoracodorsal arterial channel (TDA1), and the two thoracodorsal nerve trunks (TDN1 and TDN2) within the same thoracodorsal region. PD* identifies the posterior-cord-derived communicating branch, and the star marks its communication with an intercostobrachial/lateral cutaneous intercostal branch. (B) Further dissection demonstrating the courses and close topographic relationships of TDA1, TDA2, TDN1, and TDN2 to the surrounding axillary vessels and neural structures. PD* again identifies the posterior-cord-derived communicating branch, and the star marks the communication point. Digital enhancement was applied solely to improve visual discrimination of already visible anatomical structures. Because artery-to-nerve distances, venous anatomy, and intramuscular distributions were not mapped, the figure demonstrates close topographic association rather than two complete or functionally independent neurovascular pedicles. AA, axillary artery; BA, brachial artery; CSA, circumflex scapular artery; ICBN1–4, intercostobrachial/lateral cutaneous intercostal branches; LSSA1, lower subscapular artery; LTA1–2, lateral thoracic arteries; LTDT, lateral thoracic-thoracodorsal trunk; LTN, long thoracic nerve; PCHA, posterior circumflex humeral artery; PD*, posterior-cord-derived communicating branch; RN, radial nerve; TAA, thoracoacromial artery; TDA1, accessory thoracodorsal arterial channel; TDA2, conventional thoracodorsal artery; TDN1–2, thoracodorsal nerve trunks; USSA1–2, upper subscapular arteries; AnA, angular artery; LD-latissimus dorsi muscle. Orientation: S, superior; I, inferior; L, lateral; M, medial.

Digital enhancement was limited to color overlays and text labels applied to structures already visible in the photographs to facilitate discrimination of arterial and neural elements. No anatomical structure was added, removed, displaced, or reconstructed. Figure 1A provides an unaltered reference view of the dissection field; color-enhanced panels are identified explicitly in the figure legends.

At the proximal axillary artery, a conventional superior thoracic artery and a second arterial channel directed toward the superior thoracic region were observed. Because the caliber, complete branching pattern, and terminal distribution of the second channel were not prospectively measured, it is described here as an accessory superior thoracic arterial channel rather than as a proven true duplication of the superior thoracic artery.

We identified two arteries within the thoracodorsal region. One followed the conventional subscapular pathway and is referred to as the conventional TDA (TDA2). The second, TDA1, was traced proximally to the lateral thoracic-thoracodorsal trunk (LTDT) and arose from this trunk before descending toward the thoracodorsal region; it is therefore described as an accessory thoracodorsal arterial channel. The conventional TDA was present and was not replaced by the anomalous channel. Both channels were followed distally toward the posterior axillary wall/LD region, but their terminal intramuscular distributions were not mapped; accordingly, the manuscript does not claim two independently perfusing LD territories.

We traced two neural trunks, TDN1 and TDN2, proximally to separate origins from the PC, with TDN1 arising more proximally and TDN2 more distally. The absence of a common proximal stem distinguishes this arrangement anatomically from distal bifurcation of a single TDN. Both trunks were followed distally toward the LD as far as the preserved dissection permitted, but their intramuscular entry points and distributions were not mapped; stimulation and histology were also not performed. Thus, the observation demonstrates two independently arising TDN trunks but does not establish separate functional motor territories.

Within the exposed axilla, the two nerves and two thoracodorsal arterial channels coursed in close topographic association. However, we did not record standardized artery-to-nerve distances, and we did not map the venous anatomy. We therefore avoid designating two discrete complete neurovascular pedicles and instead describe two independently arising TDN trunks and two thoracodorsal arterial channels in close regional association.

A separate branch arose from the PC between the origins of TDN1 and TDN2 and coursed toward an intercostobrachial/lateral cutaneous intercostal branch, where we observed a macroscopic communication. Its course differed from the exposed subscapular and thoracodorsal branches because it did not follow them toward the subscapularis or LD in the dissected field and instead terminated at the communicating point. The functional fiber composition and direction of this communication cannot be determined from gross anatomy.

We retained four intercostobrachial/lateral cutaneous intercostal branches in the figure labels as ICBN1-4 to preserve orientation with the previously published dissection [12]. These numbers are positional labels and should not be interpreted as verified T2-T5 segmental origins, because the intercostal roots were not re-exposed to the thoracic wall during the present re-dissection.

## Discussion

The principal direct observation is the presence of two TDN trunks arising independently from the PC and two thoracodorsal arterial channels within the same axillary region. Separate PC origins support anatomical duplication of the neural component relative to the usual single TDN that later bifurcates; they do not, by themselves, establish two functionally independent motor supplies. Muthoka et al. described posterior-cord branching sequences containing two TDN branches [5], Pattanshetti et al. reported accessory TDNs arising from the PC [6], and Bozhikova et al. described accessory thoracodorsal innervation from the axillary nerve [7].

The recent report by Ormston and Lin is particularly relevant because both neural branches were stimulated intraoperatively and each produced LD contraction [16]. That functional demonstration is stronger than gross-anatomical separation alone. In the present cadaver, stimulation and intramuscular tracing were not available; the case should therefore be read as evidence of anatomical multiplicity at origin rather than proof of two independent functional motor territories.

The arterial finding is also best stated conservatively. Previous authors have described accessory TDAs coexisting with a conventional TDA, including bilateral accessory vessels and direct axillary origins [3,4]. An accessory TDA has classically been identified by its course, distribution, and termination in the LD territory [3]. In the present specimen, the conventional subscapular-TDA route was preserved, and the second thoracodorsal-directed channel was traced proximally to and arose from the LTDT. Because terminal intramuscular distribution and vessel calibers were not prospectively documented, we describe this as a conventional TDA with an accessory thoracodorsal arterial channel rather than claiming two independently perfusing TDAs.

For the same reason, the second vessel in the superior thoracic region is described as an accessory superior thoracic arterial channel rather than unequivocal STA duplication. Smith et al. documented a rare dual superior thoracic arterial configuration [9], but complete classification in the present specimen would require more detailed data on origin, caliber, branching, and terminal distribution than were recorded.

A major interpretive issue is the relationship to the authors' 2023 report from the same donor [12]. That report already illustrated the right axillary arterial field, one TDA accompanied by a TDN, and four intercostobrachial/lateral cutaneous branches. The present case therefore does not claim novelty for those structures. Its additional value lies in the subsequent deeper tracing that demonstrated a second PC-origin TDN, clarified the additional thoracodorsal-directed arterial pathway, and revealed the PC-intercostobrachial communication. The transparency table summarizes this distinction.

This donor was not included in the 30-cadaver/60-side series investigating intercostobrachial nerve variability reported by Piagkou et al. in 2025 [15].

The PC-intercostobrachial communication adds a distinct neural observation. Meta-analytic and cadaveric studies confirm that ICBN communications with brachial plexus branches are common, although the targets vary [10,15]. Because the communicating branch in this specimen originated from the PC and no histology or electrophysiology was performed, its fiber composition cannot be inferred. Sensory transfer, motor contribution, mixed fibers, or a persistent developmental connection remain possibilities rather than established functions.

The developmental explanation should likewise remain hypothetical. The upper-limb arterial tree develops through remodeling of an early vascular plexus, and persistence of normally regressing channels is a recognized general mechanism of arterial variation [13]. This framework could account for coexistence of conventional and accessory arterial pathways, but it does not establish the case-specific mechanism. No causal developmental link between the arterial and neural variations can be inferred from the adult specimen.

Clinically, these findings matter because unexpected additional arteries or nerve trunks may complicate identification and preservation of thoracodorsal structures during axillary lymph node dissection, breast surgery, and LD/TDAP flap harvest [1,2,8,14]. An additional arterial channel could represent a potential source of bleeding or an additional vascular route, although its actual perfusion contribution cannot be determined from the present specimen. Likewise, injury to one of two independently arising nerve trunks could theoretically affect LD innervation, but the present case does not establish the magnitude or distribution of such an effect.

Computed tomographic angiography has been investigated for preoperative TDAP perforator mapping and may provide detailed vascular anatomy and three-dimensional reconstructions, although evidence remains limited and protocols are not standardized [17]. Fine accessory channels may not be consistently detected, and the present specimen underwent no pre-dissection imaging. Accordingly, imaging is mentioned only as a possible adjunct in reconstructive planning, not as a reliable method for diagnosing the exact configuration described here.

This report has several limitations. It represents a single formalin-embalmed specimen and cannot establish prevalence. Venous anatomy was not mapped, objective morphometric measurements were not prospectively recorded, and no vascular injection or perfusion study was performed. Nerve stimulation, histology, electrophysiology, and intramuscular tracing were unavailable; therefore, functional fiber composition, separate motor territories, and functional independence cannot be determined. The segmental origins of the four intercostobrachial/lateral cutaneous branches were not re-confirmed in the present dissection. These limitations preclude classification of the anatomy as two complete and functionally independent neurovascular pedicles, but they do not alter the directly observed presence of two separate PC-origin TDN trunks and two thoracodorsal arterial channels in close regional association.

## Conclusions

This cadaveric case demonstrates two TDN trunks with separate origins from the posterior cord and two thoracodorsal arterial channels in close topographic association: a conventional subscapular TDA and an accessory thoracodorsal arterial channel arising from the lateral thoracic-thoracodorsal trunk. An accessory superior thoracic arterial channel and a communication between an intercostobrachial/lateral cutaneous intercostal branch and a PC-derived branch were also present. Because venous components, distal intramuscular territories, and functional contributions were not mapped, the anatomy should not be described as two complete, functionally independent neurovascular pedicles. The surgical implications are therefore potential rather than demonstrated: awareness of additional arterial and neural structures may help avoid inadvertent injury and incomplete preservation during axillary operations and LD/TDAP flap procedures.
